# Therapeutic drug monitoring of selumetinib in pediatrics: a combined LC-MS/MS and LC-HRMS approach 

**DOI:** 10.3389/fphar.2025.1649335

**Published:** 2025-08-18

**Authors:** Alessia Cafaro, Andrea Santangelo, Sebastiano Barco, Corinna Corsini, Roberto Bandettini, Pasquale Striano, Maria Cristina Diana, Giuliana Cangemi

**Affiliations:** ^1^ Biochemistry, Pharmacology and Newborn Screening Unit, Central Laboratory of Analysis, IRCCS Istituto Giannina Gaslini, Genoa, Italy; ^2^ Department of Neurosciences, Rehabilitation, Ophthalmology, Genetics, Maternal and Child Health, University of Genoa, Genova, Italy; ^3^ Paediatric Neurology and Muscular Disease Unit, IRCCS Istituto Giannina Gaslini, full member of ERN EpiCARE, Genoa, Italy; ^4^ Department of Chemistry and Industrial Chemistry, University of Genoa, Genoa, Italy

**Keywords:** neurofibromatosis type 1, selumetinib, therapeutic drug monitoring, LC-MS/MS, metabolites

## Abstract

Neurofibromatosis type 1 (NF1) is a genetic disorder characterized by the development of plexiform neurofibromas (PNs), benign yet potentially debilitating tumors with limited treatment options. Selumetinib, a selective MEK1/2 inhibitor, has emerged as a targeted therapy for symptomatic, inoperable PNs in pediatric NF1 patients. Individual variability in drug metabolism, largely influenced by CYP450-mediated pathways, can affect treatment response. In this study, we describe a novel liquid chromatography–tandem mass spectrometry (LC–MS/MS) method for the quantification of selumetinib in human plasma. The method was validated in accordance with ICH M10 guidelines in the range 1.3–2,000 ng/mL and demonstrated high selectivity, precision and accuracy. Its clinical applicability was assessed in pediatric NF1 patients receiving selumetinib, with measured Ctrough levels ranging from 15.80 to 537.39 ng/mL. To further investigate interindividual pharmacokinetic variability, we applied liquid chromatography–high-resolution mass spectrometry (LC–HRMS) to profile selumetinib metabolites. A total of ten metabolites were identified, including the pharmacologically active N-desmethyl-selumetinib (M8). Metabolite-to-parent ratios (MPRs) suggested notable interpatient differences in metabolic patterns. This combined LC–MS/MS and LC–HRMS strategy provides both precise quantification of selumetinib and insight into patient-specific metabolic profiles. Beyond its analytical strengths, the approach supports therapeutic drug monitoring (TDM) and paves the way for personalized selumetinib dosing.

## 1 Introduction

Neurofibromatosis type 1 (NF1) is an autosomal dominant neurocutaneous disorder caused by variants in the *NF1* gene, which encodes for neurofibromin, a RAS GTPase-activating protein that negatively regulates the MAPK/MEK pathway. Its incidence is ∼1 in 2,500–3,000 individuals (Williams et al., 2009; Shen et al., 1996). The loss-of-function mutations of *NF1* gene lead to uncontrolled cell proliferation and growth (Anderson et al., 2021). One of the most common complications are plexiform neurofibromas (PNs), affecting 30%–50% of patients (Huson et al., 1989; Tonsgard, 2006). These benign tumors originate from Schwann cells and involve perineural cells, fibroblasts, and mast cells, forming a heterogeneous microenvironment (Hirbe and Gutmann). Although PNs are non-malignant, they can grow rapidly, especially in early childhood, causing disfigurement and compression of vital structures (van Noesel et al., 2019; Nguyen et al., 2011). Furthermore, PNs carry a 15% lifetime risk of progressing to malignant peripheral nerve sheath tumors (MPNSTs) (Legius et al., 2021; Gross et al., 2018; Ferrari et al., 2011).

Traditionally, surgery has been the primary treatment for PNs. However, its effectiveness is limited by the complex integration of these tumors within nerve plexuses and their high vascularity, which heightens the risk of complications and recurrence. Furthermore, nearly 50% of PNs are deemed inoperable (Anderson et al., 2021).

Selumetinib is a potent, selective, orally administered small-molecule inhibitor that noncompetitively blocks ATP binding to MEK1 and MEK2, preventing their phosphorylation. By binding to MEK1/2, selumetinib induces conformational changes that disrupt intracellular signal transduction, ultimately inhibiting tumor cell growth and proliferation (Campagne et al., 2020). Selumetinib has been approved for the treatment of symptomatic, inoperable PNs in pediatric patients with NF1 aged 3 years and older (Koselugo, 2025). Selumetinib has revolutionized the treatment landscape for symptomatic NF1-associated tumors (Sanchez et al., 2021; Souza et al., 2022; Solares et al., 2021). However, durable response rates have been observed in 56% of patients, as assessed through a combination of volumetric radiographic measurements and quality of life scales (Gross et al., 2020). Variations in treatment response can be partially explained by the fact that PNs exhibit distinct genetic signatures associated with sensitivity to selumetinib (Bhandarkar et al., 2024).

Selumetinib undergoes hepatic metabolism via CYP3A4-mediated phase I oxidation, with minor contributions from CYP2C19, CYP1A2, CYP2C9, CYP2E1, and CYP3A5. Phase II glucuronidation is catalyzed by UGT1A1 and UGT1A3 (Dymond et al., 2016). Its metabolism produces a total of 15 metabolites, including N-desmethyl-selumetinib (M8), which is 3–5 times more potent and accounts for 20%–30% of the drug’s activity (Anderson et al., 2021; Campagne et al., 2020; Koselugo, 2025). Its formation accounts for ∼10–11% of selumetinib metabolism, with a metabolite-to-parent ratio (MPR) of 5%–15% (Campagne et al., 2020). Genetic variability in biotransformation enzymes can significantly impact the pharmacokinetics (PK) and clinical response to drugs, leading to differences in drug efficacy and safety. This variability may result in altered responses to standard drug doses, increasing the risk of therapeutic failure or adverse drug reactions (Dymond et al., 2017). Due to its hepatic metabolism via CYP450 enzymes, selumetinib exposure may be influenced by genetic polymorphisms in metabolizing enzyme genes (Dymond et al., 2017). Therapeutic Drug Monitoring (TDM), through the measurement of selumetinib levels in blood or plasma, could serve as a valuable tool for the early detection of altered drug exposure. Additionally, monitoring metabolite levels and calculating the MPR can provide a direct assessment of enzymatic activity, enabling enzyme phenotyping of CYP-mediated metabolism.

In this paper, we present the development and validation of a bioanalytical method based on liquid chromatography coupled with tandem mass spectrometry (LC–MS/MS), along with its application to clinical samples from pediatric NF1 patients with PNs. In parallel, we explore the metabolic profile of selumetinib using liquid chromatography–high-resolution mass spectrometry (LC–HRMS). This integrated LC–MS/MS and LC–HRMS approach enables both accurate quantification of selumetinib and the characterization of individual metabolic patterns. The strategy holds strong clinical potential by paving the way for TDM and personalized selumetinib dosing. However, as this is a pilot study, the findings should be interpreted with caution and confirmed in larger patient cohorts.

## 2 Materials and methods

### 2.1 Chemicals

All chemicals used were of high purity (≥98%). Formic acid (99.9%), LC–MS/MS-grade Acetonitrile (ACN), zinc sulfate heptahydrate, and dimethyl sulfoxide were obtained from Sigma-Aldrich Srl (Milan, Italy). LC–MS/MS-grade methanol came from Carlo Erba Reagents (Cornaredo, Milan, Italy). Milli-Q water (MS grade) was produced using a Milli-DI system with a Synergy 185 unit (Millipore, Milan, Italy). HPLC mobile phases were filtered through 0.45 µm Millipore membrane filters (Millipore, Vimodrone, Italy). Selumetinib (C4492) and its isotopically labeled internal standard (IS) [13C2,2H4]-Selumetinib (C4493) were purchased from Alsachim (Illkirch Graffenstaden, France).

### 2.2 Stock and working solutions

Selumetinib stock solution (5 mg/mL) was prepared by dissolving 5 mg of powdered selumetinib in 1 mL of DMSO in a glass vial. A selumetinib 100 μg/mL working solution (WS1) was obtained by diluting the stock solution 1:50 (10 µL of stock solution + 490 µL of DMSO). A second working solution (WS2, selumetinib 1 μg/mL) was prepared by further diluting WS1 1:100 (10 µL of WS1 + 990 µL of DMSO). Internal standard (IS) stock solution (1 mg/mL) was prepared by dissolving 1 mg of powdered [13C2,2H4]-Selumetinib in 1 mL of DMSO in a glass vial. A 100 μg/mL IS working solution (IS WS1) was obtained by diluting the IS stock solution 1:10 (50 µL of stock + 450 µL of DMSO). A second 1 μg/mL IS working solution (IS WS2) was prepared by further diluting IS WS1 1:100 (10 µL of IS WS1 + 990 µL of DMSO). All solutions were stored at −20°C.

### 2.3 Calibration standard and quality controls

Calibration and quality control (QC) samples were prepared by spiking blank plasma with different lots of selumetinib WS1 (100 μg/mL). A nine-point calibration curve was generated with the following concentrations: 1.3, 3.3, 8.2, 20.5, 51.2, 128, 320, 800, and 2,000 ng/mL. The highest calibration sample was obtained by diluting selumetinib WS1 (100 μg/mL) 1:50 in plasma (10 µL of WS1 + 490 µL of blank plasma). Subsequent calibration levels were prepared by serial dilution with a 2.5-fold dilution factor. The lower limit of quantification (LLOQ) was set at 1.3 ng/mL and the upper limit of quantification (ULOQ) at 2,000 ng/mL. QCs were prepared at the following concentrations: 4 ng/mL (QC I), 80 ng/mL (QC II), 1,600 ng/mL (QC III). QC III was prepared by diluting selumetinib WS1 (100 μg/mL) 1:62.5 in plasma (8 µL of WS1 + 492 µL of blank plasma). QC II and QC I were prepared by serial dilution from with a 20-fold dilution factor. All calibration and QC samples were divided into 50 µL aliquots and stored at −20°C.

### 2.4 Sample preparation

Each calibration standard, QC, and patient sample (50 µL) was mixed with 5 µL of IS WS2 (1 μg/mL), followed by protein precipitation using 450 µL of ACN containing 0.1% v/v formic acid (FA). The samples were subsequently centrifuged at 20,000 × g for 5 min at 4°C, and the supernatants were transferred to autosampler vials for LC–MS/MS analysis.

### 2.5 LC-MS/MS method conditions and validation

The LC-MS/MS analyses were conducted at the Giannina Gaslini Institute using a Vanquish UHPLC system coupled to a TSQ Altis Plus Triple Quadrupole mass spectrometer (Thermo Fisher Scientific, Milan, Italy). The chromatographic separation was carried out on a Thermo Scientific Accucore Polar Premium column (50 mm × 2 mm, i.d. 2.6 mm) maintained at 40°C. The mobile phase consisted of water with 0.1% v/v formic acid (Phase A) and acetonitrile with 0.1% v/v formic acid (Phase B), pumped at a flow rate of 500 μL/min. The total runtime was 3.5 min and the gradient elution is detailed in Table 1. The injection volume was 2 µL. MS/MS detection was performed using an electrospray ionization (ESI) source in positive ion mode for both selumetinib and its IS. The ionization was achieved with a spray voltage of 3500 V, with nitrogen (99.9%) was employed as the sheath (40 arbitrary units) and auxiliary gas (15 arbitrary units). The ion transfer tube and vaporizer were both set to 350°C. Argon (99.9%) was used as the collision gas at a pressure of 1.5 mTorr. The most intense precursor ions formed in the ESI source were selected to maximize signal-to-noise ratio (S/N). The multiple reaction monitoring (MRM) transitions were: 456.9 → 394.9 for selumetinib and 463.1 → 394.9 for its IS.

**TABLE 1:** Gradient elution conditions.

Time (min)	Flow (µL/min)	Phase A %	Phase B %
0.00	500	80	20
0.10	500	80	20
1.30	500	0	100
2.50	500	0	100
2.50	500	80	20
3.50	500	80	20

The LC–MS*/*MS method was fully validated according to the ICH guidelines M10 (ICH M10 on bioanalytical method validation - Scientific guideline | European Medicines Agency, 2024) by evaluating: selectivity, matrix effect, extraction recovery, linearity, precision and accuracy, carryover, and stability.

Selectivity was assessed using plasma samples from six healthy donors. Each lot was analyzed both as unspiked and spiked with selumetinib at the LLOQ (1.3 ng/mL). Interference from endogenous compounds was considered acceptable if the response in unspiked samples did not exceed 20% of the LLOQ signal for selumetinib and 5% of the IS response.

The matrix effect (ME) on selumetinib analysis was evaluated by analyzing three replicates of QC I (4.00 ng/mL) and QC III (1,600 ng/mL), each prepared using plasma from six different individual sources. ME was determined by comparing the chromatographic peak area of selumetinib spiked after extraction with that of a pure selumetinib solution at the same concentration, using the following formula:

$$ME\%=\frac{Peak\;area\;\left(post-extraction\right)}{Peak\;area\;\left(Pure\;standard\right)}\;*\;100$$

Selumetinib extraction recovery (ER) was investigated by analyzing three replicates of QC I (4.00 ng/mL) and QC III (1,600 ng/mL), each prepared using plasma from six different individual sources. ER was calculated as the ratio of the chromatographic peak area of selumetinib spiked before extraction to that of selumetinib spiked after extraction, using the following formula:

$$ER\%=\frac{Peak\;area\;\left(post-extraction\right)}{Peak\;area\;\left(Pre-extraction\right)}\;*\;100$$

The calibration curve was prepared over a concentration range of 1.3–2,000 ng/mL and assessed for linearity by analyzing it in triplicate. The ratio of the selumetinib peak area to the IS peak area was plotted against the nominal concentrations using a 1/x weighting factor. Accuracy was considered acceptable if the calculated concentrations were within ±20% for the LLOQ and ±15% for all other calibration levels.

LLOQ, QC I, QC II, and QC III were analyzed in quintuplicate to evaluate intra-run accuracy and precision and in triplicate to assess inter-run accuracy and precision. The results were considered acceptable if they fell within ±15% for the QCs and ±20% for the LLOQ.

Carryover was evaluated by analyzing three blank samples following the injection of the highest calibration standard (selumetinib, 2,000 ng/mL). It was considered negligible if the signal remained within ±20% of the LLOQ signal for selumetinib and ±5% for the IS.

The stability of extracted samples in the autosampler was evaluated by re-analyzing them after 24 h. The stability of selumetinib in plasma was assessed analyzing QC I and QC III stored in a refrigerator at 4°C ± 3°C, and in a freezer at −20°C. Samples were analyzed at the following time points: 0, 7, 14, and 30 days, with each analysis performed in triplicate. Selumetinib was considered stable if the deviation from the 0-day concentration remained within ±15%. Selumetinib stock solutions were aliquoted and stored at −20°C. Long-term stability was evaluated after 1 and 2 months, while freeze–thaw stability was assessed over three cycles. Bench-top stability was tested at room temperature for up to 6 h. Recoveries were calculated relative to freshly prepared solutions.

### 2.6 LC-HRMS conditions

LC-HRMS analyses were carried out using a UHPLC Vanquish Transcend Duo system coupled to a Orbitrap Exploris 120 Mass Spectrometer (Thermo Fisher Scientific, Milan, Italy). Chromatographic separation was performed using a Waters ACQUITY UPLC CSH C18 Column (130 Å, 1.7 µm, 2.1 mm × 100 mm, 1/pk), maintained at 45°C. The mobile phases consisted of water with 0.1% v/v formic acid (phase A) and ACN with 0.1% v/v formic acid (phase B), with a flow rate of 300 μL/min. The gradient started with 1% phase B, increasing to 99% over 10 min, and was held at 99% for 5 min. Subsequently, the proportion of phase B was reduced to 1% within 1 min, followed by a column wash at 1% phase B for 4 min, resulting in a total runtime of 20 min.

Ionization was achieved using an ESI source in both positive (3500 V) and negative (2500 V) modes. Nitrogen was used as the sheath and auxiliary gases, set at 50 and 10 arbitrary units, respectively. The ion transfer tube and vaporizer temperatures were set to 325°C and 350°C. Data were acquired in MS Full Scan mode at a resolution of 120,000 (mass range 100–1,000 m/z). The raw data files were processed using Compound Discoverer software, which also enabled the simulation of Phase I and Phase II selumetinib metabolic reactions. Ionic currents corresponding to putative m/z values were extracted and filtered based on peak chromatographic quality. The mass spectra of the putative metabolites were compared to those of pure selumetinib standards, selecting metabolites with a fragmentation pattern match score above 80%. Since selumetinib contains a chlorine atom, the natural isotopic distribution of chlorine (35Cl and 37Cl) was used to confirm the identity of the metabolite. In particular, the detection of both isotopologues provided additional evidence supporting the correct structural assignment of the metabolite.

### 2.7 Clinical samples

The study was conducted in accordance with the ethical standards of the Institutional and National Research Committee and the 1975 Helsinki Declaration, as revised in 2013. Written informed consent was obtained from all patients or their legal representatives upon admission, allowing the use of clinical data for research purposes in compliance with the privacy policy of IRCCS Istituto Giannina Gaslini, Genoa, Italy. Clinical validation of the LC-MS/MS method was carried out using 16 pre-dose (C_trough_) and two post-dose plasma samples from pediatric patients with NF1 receiving selumetinib at a dose of 25 mg/m^2^ twice daily (BID) for inoperable PNs. Post-dose samples were collected following the morning administration. Additionally, LC-HRMS analyses were performed on the same samples to assess the selumetinib metabolic profile and MPR. Peripheral blood samples were obtained by venipuncture and collected into K_3_EDTA tubes. Following centrifugation at 4,000 × g for 5 min, plasma was separated and stored at −20°C until analysis.

## 3 Results

### 3.1 LC-MS/MS method validation

**FIGURE 1:**
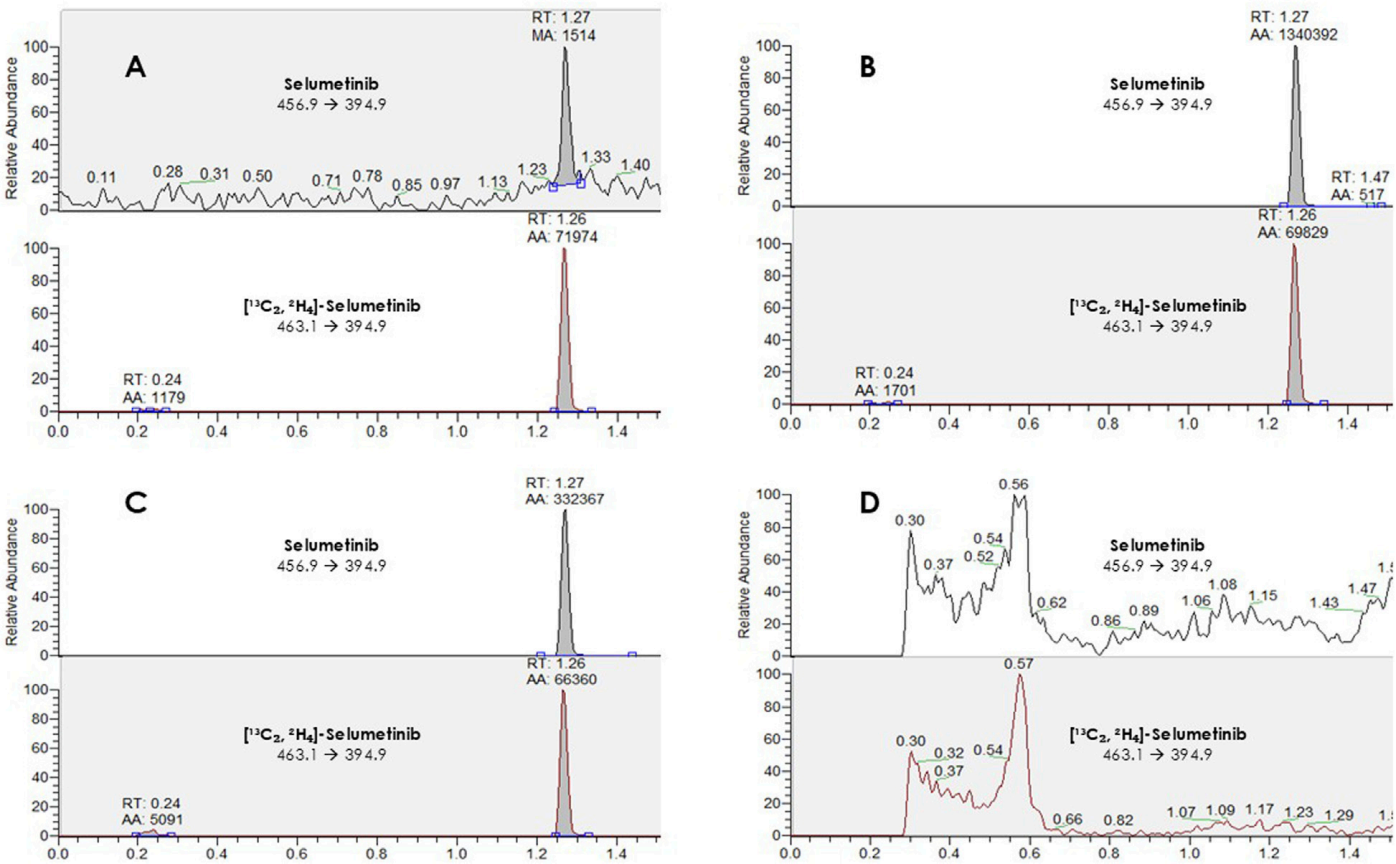
Chromatographic peaks obtained from a lower limit of quantification (LLOQ) sample **(A)**, an upper limit of quantification (ULOQ) sample **(B)**, a patient sample **(C)**, and a blank sample **(D)**.

**FIGURE 2:**
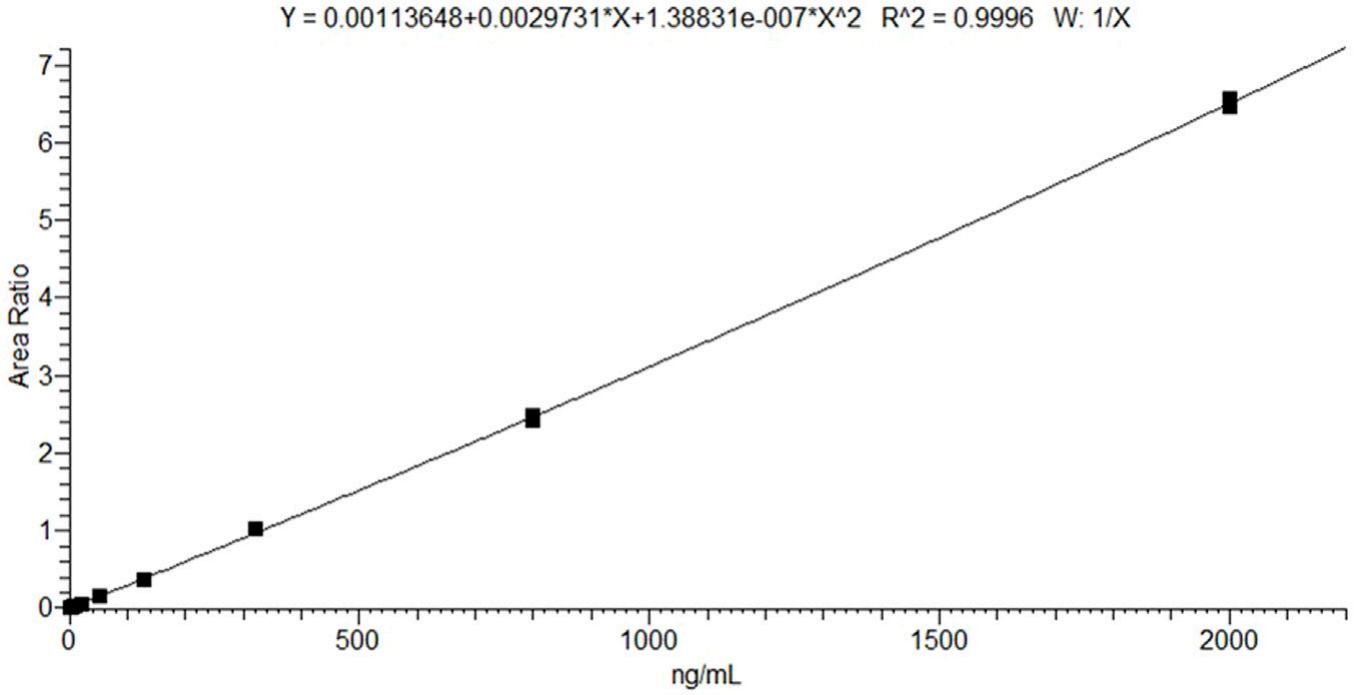
Mean 9-point calibration curve (*n* = 3) in the concentration range 1.3–2,000 ng/mL.

The LC–MS/MS method provided efficient chromatographic separation and well-defined peak shapes for selumetinib (Figures 1A–C). The mean retention time was 1.27 ± 0.10 min. No interfering peaks were observed at the selumetinib retention time across six different lots of blank plasma samples (Figure 1D). The method met the acceptance criteria outlined in the ICH M10 guidelines for ME, ER, precision, and accuracy (Table 2). Validation was conducted over a calibration range of 1.3–2,000 ng/mL. The calibration curve for selumetinib was generated using quadratic regression, plotting the selumetinib-to-IS peak area ratio with a 1/x weighting factor (Figure 2). The method exhibited excellent linearity across the entire concentration range, with an *R* ^2^ value of 0.99. Back-calculated concentrations for all analytes were within ±15% of nominal values. Carryover was negligible. Selumetinib resulted stable in plasma samples for up to 30 days when stored at 4°C ± 3°C in a refrigerator and at −20°C in a freezer (percentage difference within ±15%). Selumetinib stock solutions in DMSO were stable for up to 2 months at −20°C, with recoveries ≥98%. After three freeze–thaw cycles, recovery remained ≥96% with minimal degradation. Bench-top stability tests showed ≥97% recovery after 6 h at room temperature.

**TABLE 2:** Results from ME, ER, precision and accuracy validation experiments.

Matrix effect and extraction recovery (*n* = 3)		
QC level	ME%	ER%
QC I	114%	110%
QC III	94%	95%

Intra-run precision and accuracy (*n* = 5)				
QC level	Nominal concentration (ng/mL)	Mean measured concentration ± SD (ng/mL)	CV%	Accuracy %
LLOQ	1.3	1.26 ± 0.22	18%	97%
QC I	4	4.07 ± 0.39	10%	102%
QC II	80	78.32 ± 1.07	1%	102%
QC III	1,600	1688.60 ± 95.89	6%	106%

Inter-run precision and accuracy (*n* = 3)				
LLOQ	1.3	1.42 ± 0.07	5%	109%
QC I	4	4.16 ± 0.45	11%	104%
QC II	80	77.37 ± 8.62	11%	97%
QC III	1,600	1642.99 ± 69.17	4%	103%

SD is standard deviation.

### 3.2 Clinical samples

Patients were both males (n. 11) and females (n. 7) with a median age of 14.9 years (IQR, 5.5). The median treatment duration was 29.3 months (IQR, 31.8), during this time no patient underwent treatment suspension, and all included patients presented mild to moderate cutaneous side effects. The mean observed C_trough_ in real samples was 119.54 ng/mL (standard deviation, SD 134.54 ng/mL), with concentrations in pediatric patients ranged between 15.80 and 537.39 ng/mL. The two post-dose concentration resulted 609.50 and 537.67 ng/mL.

**FIGURE 3:**
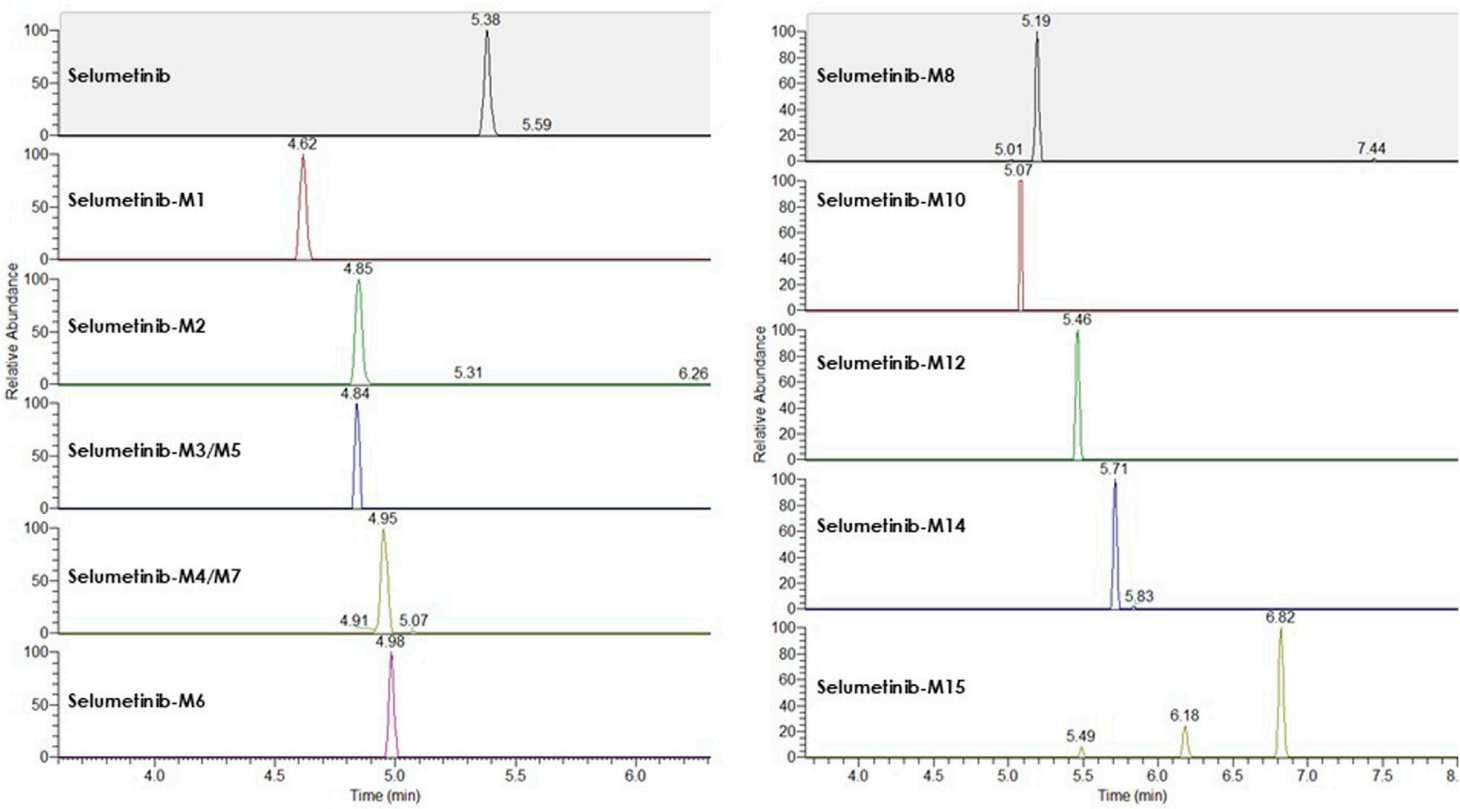
Representative LC-HRMS chromatograms of selumetinib and its detected metabolites obtained from a pooled sample created by combining plasma from multiple patients.

**FIGURE 4:**
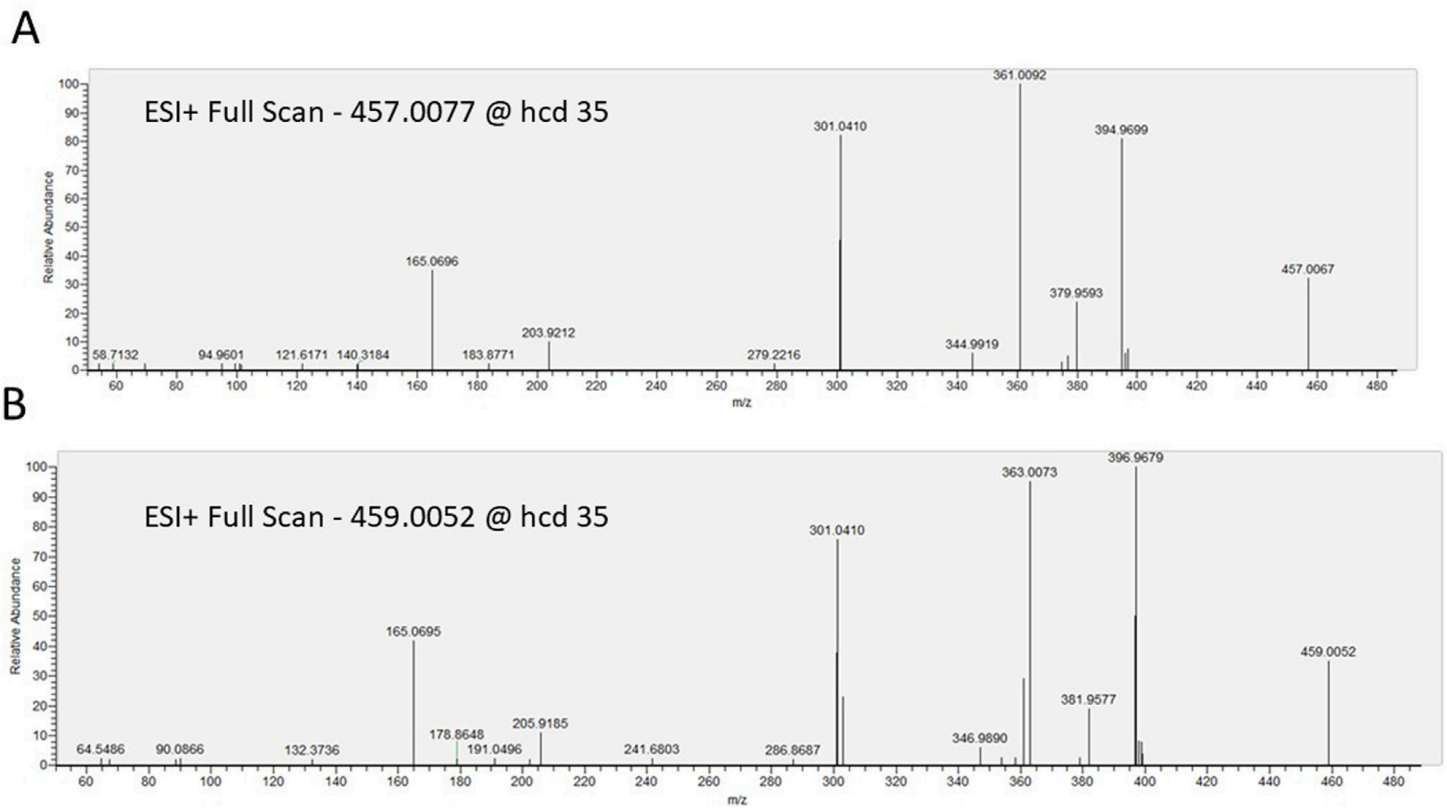
Representative fragmentation spectra of selumetinib with chlorine isotopes Cl^−35^ **(A)** and Cl^−37^ **(B)**, acquired by higher-energy collisional dissociation (HCD) at a normalized collision energy of 35.

LC-HRMS analysis enabled the detection of several selumetinib metabolites, including M1, M2, M3/M5, M4/M7, M6, M8, M10, M12, M14, and M15. Metabolites M9, M11, and M13 were not detected. Chromatographic peaks corresponding to either M3 or M5, and to M4 or M7, were observed; however, distinction between these isomers was not possible in the absence of authentic standards. Figure 3 illustrates representative chromatograms of selumetinib and its metabolites. Detailed information on the molecular formulas, theoretical masses, and corresponding m/z values for each metabolite is reported in Table 3. The higher-energy collisional dissociation (HCD) fragmentation spectra of selumetinib, which support metabolite identification, are shown in Figure 4. MPR values, calculated as the ratio of each metabolite’s peak area to that of selumetinib, are summarized in Table 4.

**TABLE 3:** Molecular formulas, theoretical monoisotopic masses (based on the Cl-35 isotope) and corresponding [M+H]^+^ m/z values for selumetinib and its detected metabolites.

Metabolite	Molecular formula	Theoretical mass	Theoretical m/z	Detected m/z	Mass error (ppm)
Selumetinib	C_17_H_15_BrClFN_4_O_3_	456.0000	457.0073	457.0074	0.22
Selumetinib-M1	C_15_H_10_BrClFN_3_O_2_	555.9797	556.9869	556.9875	1.08
Selumetinib-M2	C_21_H_17_BrClFN_4_O_7_	569.9953	571.0026	571.0037	1.93
Selumetinib-M3/M5	C_22_H_21_BrClFN_4_O_9_	618.0164	619.0237	619.0245	1.29
Selumetinib-M4/M7	C_23_H_23_BrClFN_4_O_9_	632.0321	633.0394	633.0404	1.58
Selumetinib-M6	C_21_H_17_BrClFN_4_O_8_	585.9902	586.9975	586.9900	−12.78
Selumetinib-M8	C_16_H_13_BrClFN_4_O_3_	441.9844	442.9916	442.9923	1.58
Selumetinib-M10	C_14_H_7_BrClFN_4_O	379.9476	380.9549	380.9557	2.10
Selumetinib-M12	C_14_H_9_BrClFN_4_O	381.9632	382.9705	382.9706	0.26
Selumetinib-M14	C_15_H_11_BrClFN_4_O	395.9789	396.9862	396.9862	0.00
Selumetinib-M15	C_15_H_10_BrClFN_3_O_2_	396.9629	397.9702	397.9721	4.77

Mass errors are expressed in parts per million (ppm).

**TABLE 4:** The ratio of metabolite-to-parent drug % (MPR %) for selumetinib and main metabolites detected in patients’ samples.

Statistic	M1%	M2%	M3/M5%	M4/M7%	M6%	M8%	M10%	M12%	M15%	M14%
Mean	2%	30%	0%	3%	0%	7%	0%	2%	14%	5%
SD	2%	16%	0%	2%	0%	2%	0%	2%	8%	3%
Median	2%	26%	0%	2%	0%	6%	0%	2%	14%	4%
Min	0%	15%	0%	0%	0%	3%	0%	0%	1%	1%
Max	10%	78%	2%	7%	1%	12%	2%	8%	26%	11%

SD is standard deviation.

Selumetinib was the predominant circulating drug-related compound, accounting for 68.2% of the total signal, followed by M2 (14.5%), M15 (7.45%), M8 (3.49%), M14 (1.92%), M12 (1.25%), M4/M7 (1.8%), M1 (0.85%), M3/M5 (0.27%), M6 (0.15%), and M10 (0.12%).

**FIGURE 5:**
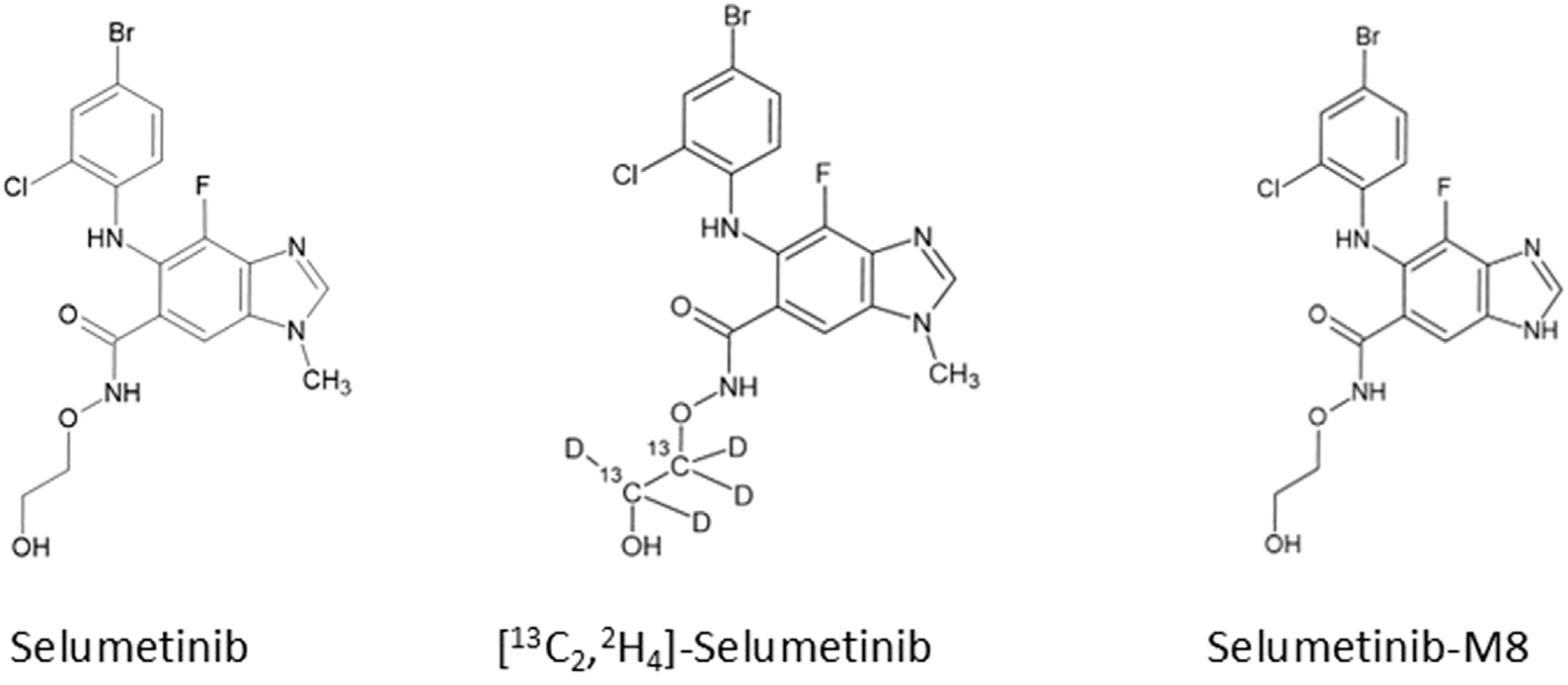
Chemical structures of selumetinib, its internal standard ([^13^C_2_,^2^H_4_]-selumetinib), and the active metabolite M8.

Chemical structures of selumetinib, its IS [^13^C_2_,^2^H_4_]-selumetinib, and the active metabolite M8 are shown in Figure 5.

## 4 Discussion

This paper describes the development and validation of a robust LC-MS/MS method for quantifying selumetinib in plasma microsamples (50 µL), alongside a semiquantitative metabolic profile obtained via LC-HRMS.

Although a few LC-MS/MS methods have been previously reported (Voggu et al., 2020; Severin et al., 2016), this is the first applied to real-life samples from pediatric patients. The proposed method offers several advantages, including a short run time (3.5 min), a low LLOQ (1.3 ng/mL), and a simplified sample preparation without the need for nitrogen drying. Furthermore, it enables the quantification of selumetinib from a small plasma volume (50 µL), which is particularly advantageous in pediatric clinical settings where blood collection is often challenging. The stability of selumetinib in solutions and plasma samples has been demonstrated for up to 2 months and 1 month, respectively, and the results are consistent with previously published studies (Voggu et al., 2020; Severin et al., 2016; Borale et al., 2024).

Trough selumetinib concentrations showed marked interindividual variability, ranging from 15.80 to 537.39 ng/mL. Although TDM is not yet part of routine clinical practice for selumetinib therapy, these findings support its potential utility in optimizing treatment. Further real-world PK studies are warranted to better elucidate the link between drug exposure, clinical response, and toxicity.

From a clinical perspective, MPR analysis could serve as a useful tool for assessing CYP3A4/5 activity in patients treated with selumetinib. Given that its metabolism is susceptible to CYP3A modulation, monitoring MPR values may help personalize therapy by identifying patients at risk of altered drug exposure due to co-medications or genetic polymorphisms affecting CYP3A4/5 function. Moreover, evaluating MPR values over time may also serve as a surrogate marker of treatment adherence, as persistently low levels of both parent drug and metabolites could reflect suboptimal intake. Additionally, interindividual differences in metabolite-to-parent ratios may correlate with the risk of adverse effects, particularly in patients with excessive formation of active or toxic metabolites. Finally, metabolic profiling could support the identification of exposure–response relationships, offering insights into whether specific metabolic patterns are associated with greater reductions in tumor volume or improved clinical outcomes. Metabolites can be simultaneously measured alongside selumetinib in biological matrices using LC-MS/MS techniques, but it requires the generation of calibration curves for each metabolite, necessitating high-purity reference standards, which can be prohibitively expensive and not available for all metabolites. HRMS offers an alternative approach, enabling metabolite detection without the need for reference standards and providing a semi-quantitative metabolic profile (Penner et al., 2013).

The metabolic profile observed in our pediatric NF1 cohort shows notable differences from that reported by Dymond et al. (2016) in healthy adults. In both studies, M2 was the main circulating metabolite, but its relative abundance was lower in our cohort (14.5% vs. 22%), possibly reflecting age- or disease-related differences in metabolism. Selumetinib-M1, M3/M5, M4/M7, and M6 were detected at 0.85%, 0.27%, 1.8%, and 0.15%, respectively, compared to 4%, 3%, 4%, and <1%, respectively, reported by Dymond et al. (2017).

The active metabolite M8 was present at 3.5%, aligning closely with the 3% reported previously. M9 and M13 were absent from both datasets, suggesting they may not circulate in plasma. A difference was observed in the proportion of unchanged selumetinib, which accounted for 68.2% of the total drug-related material in our study versus 40% in Dymond et al. This discrepancy may be due to differences in sampling time or population characteristics (pediatric NF1 patients vs. healthy adults).

This study represents a pilot investigation, and the results will need to be confirmed and expanded in a larger patient cohort.

## 5 Conclusion

This study presents a robust, sensitive, and selective LC-MS/MS method for the quantification of selumetinib in human plasma, fully validated in accordance with ICH guidelines M10. Its successful application to clinical samples from pediatric NF1 patients with inoperable PNs confirms its suitability for TDM. The complementary LC-HRMS analysis enabled detailed characterization of selumetinib’s metabolic profile, identifying major and minor metabolites and calculating individual metabolite-to-parent ratios. These findings support the potential utility of metabolic profiling for assessing interindividual variability in selumetinib exposure, paving the way for personalized therapeutic strategies in NF1 treatment.

## Data Availability

The raw data supporting the conclusions of this article will be made available by the authors, without undue reservation.
